# Heterotopic ossification of the fibula flap pedicle causing trismus in a pediatric maxillary reconstruction

**DOI:** 10.1093/jscr/rjaf1078

**Published:** 2026-01-20

**Authors:** Adam A Karkoutli, Jacob Beiriger, Hilary McCrary

**Affiliations:** Department of Otolaryngology – Head and Neck Surgery, University of Utah, Salt Lake City, UT, United States; Department of Otolaryngology – Head and Neck Surgery, University of Utah, Salt Lake City, UT, United States; Department of Otolaryngology – Head and Neck Surgery, University of Utah, Salt Lake City, UT, United States

**Keywords:** heterotopic ossification, fibula osteocutaneous free flap, pediatric maxillary reconstruction

## Abstract

We present the case of a 13-year-old boy who developed heterotopic ossification along the pedicle of a fibula osteocutaneous free flap used for complex midface reconstruction following a ballistic injury. While rare in pediatric patients, this complication resulted in severe trismus, which required surgical excision of the pedicle. This case highlights the importance of long-term surveillance and periosteal management strategies in children undergoing fibula flap reconstruction of the maxilla.

## Introduction

Heterotopic ossification of the vascular pedicle is a recognized but uncommon complication following fibula osteocutaneous free flap reconstruction in pediatric and adult patients. Heterotopic ossification is most frequently associated with retention of periosteum along the vascular pedicle. The periosteum maintains osteogenic potential and can lead to new bone formation. In pediatric patients, this may be accentuated due to higher osteogenic activity and ongoing craniofacial growth. Most pedicle ossification cases are asymptomatic and detected incidentally on imaging. Symptomatic cases can present with trismus, ankylosis, pain, or hard swelling [1–4].

In this report, we describe a case of pedicle ossification following fibula flap reconstruction of the maxilla in a 13-year-old boy after a ballistic injury. The ossified pedicle resulted in progressive trismus and required surgical debridement. This is one of the youngest patients ever reported with this complication. Pedicle ossification is a rare but important complication of fibula free flap reconstruction in children.

## Case presentation

A 13-year-old boy sustained a gunshot to the face resulting in complex maxillofacial trauma, including near-complete palatal destruction and comminuted midface fractures. He was referred for definitive reconstruction following initial stabilization and debridement.

The patient underwent extensive reconstruction two months after the injury. This included a left fibula osteocutaneous free flap for maxillary and palatal reconstruction with immediate placement of osseointegrated dental implants, as well as midface hardware fixation. The vascular pedicle was tunneled through the left cheek to reach recipient vessels in the neck. His postoperative course was complicated by the development of a 1–2 cm oroantral fistula along the reconstructed palate. This was initially managed with revision palatoplasty and a rotational local flap; however, the fistula persisted. Definitive closure was achieved using a left radial forearm free flap for mucosal resurfacing. The patient had an uncomplicated recovery and was tolerating an oral diet at his one-year follow-up. His oral hygiene, speech, and facial cosmesis were satisfactory, and plans were underway for dental prosthetic rehabilitation.

**Figure 1:**
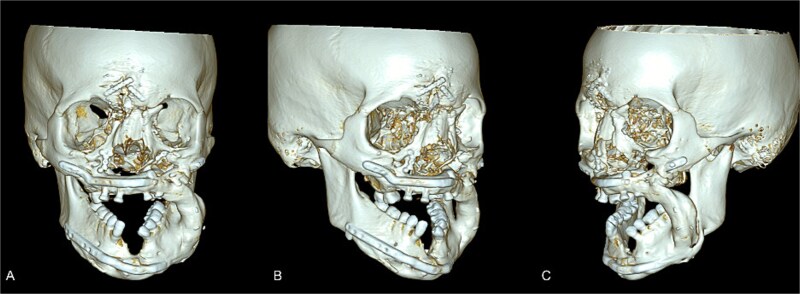
Maxillofacial CT with 3-D volume after fibula free flap. The reconstructed left midface/mandible with fibula free-flap hardware is shown with resection margins and the osseous bridge. (A) Frontal view. (B) Right anterior view. (C) Left anterior view.

**Figure 2:**
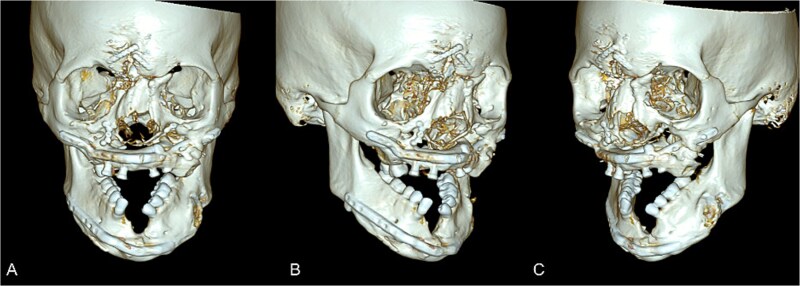
Postoperative maxillofacial CT with 3-D volume following revision surgery. The reconstructed left midface/mandible with is shown after removal of the osseous bridge. (A) Frontal view. (B) Right anterior view. (C) Left anterior view.

**Figure 3:**
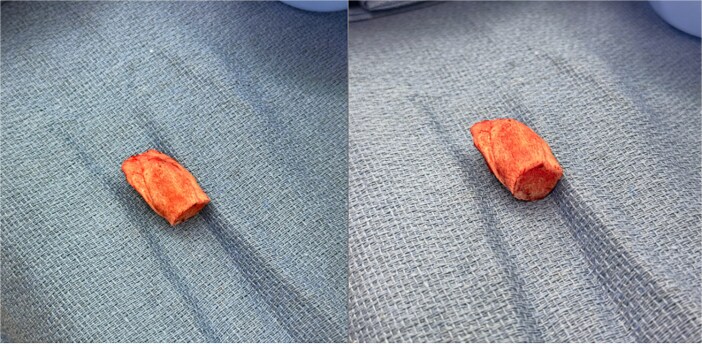
Intraoperative specimen of the osseous bridge excised en bloc from along the fibula free-flap pedicle. Photographs show the same specimen in two orientations with with heterotopic ossification.

During preoperative evaluation by the oral and maxillofacial surgery team, he was noted to have progressive and severe trismus. Despite preserved oral intake, he reported increasing difficulty with jaw opening. A computed tomography scan was obtained, which demonstrated a large tract of heterotopic ossification along the fibula flap pedicle, forming a bony bridge between the residual left posterior maxilla and the mandible (Fig. 1). The patient was scheduled for surgical intervention and underwent left facial and neck exploration with excision of a bony bridge along the fibula free-flap pedicle, intraoral adjacent tissue transfer, and left neck scar revision. A 3 cm osseous bridge was found spanning the prior fibula flap pedicle to the left maxilla/mandible and was excised (Figs 2 and 3). The resection created an 8 × 5 cm intraoral mucosal defect that was reconstructed with adjacent tissue transfer. He developed a seroma postoperatively that was drained in clinic and resolved. At most recent follow-up the neck incision was well-healed without drainage or fluid collection, and he was tolerating oral intake.

## Discussion

The fibula free flap is a well-established technique for reconstruction of extensive maxillofacial defects. Fibula free flaps offer sizeable, vascularized bone that is suitable for facial structure, dental implantation, and contour. Application in children is less frequent than adults and carries unique considerations such as growth potential, long-term follow-up, and complications that may evolve over time.

One rare complication is heterotopic ossification of the vascular pedicle. It is hypothesized that heterotopic ossification results from retained periosteum along the vascular pedicle, which has osteogenic potential when transplanted into a well-vascularized environment. Other compounding risks include mechanical stress on the pedicle, direct contact between the pedicle and native bone, and possible hormonal influences from the bloodstream [2, 5, 6]. Although this phenomenon is often asymptomatic and incidentally discovered on imaging, it can cause significant functional impairment when bridging occurs between fixed and mobile skeletal structures [7, 8].

In this patient, the development of progressive trismus one year following fibula flap maxillary reconstruction was attributed to ossification along the pedicle which had formed a bony bridge between the residual maxilla and mandible. Maxillary reconstructions may be more prone to this complication due to the superior and tensioned course of the pedicle through the cheek due to motion and strain promoting osteogenesis [7, 8]. CT imaging is the modality of choice to delineate the extent and maturity of the ossified tract.

Pediatric cases of heterotopic ossification are incredibly rare. Smith reported a similar case in a 12-year-old girl who developed severe trismus and extracapsular ankylosis due to periosteal ossification after fibula flap maxillary reconstruction [5]. Pediatric patients may be at a greater risk than adults due to higher periosteal activity, elevated hormonal factors, and more active bone remodeling during growth.

Management strategies depend on symptomatology. Asymptomatic cases may be observed, but patients with trismus, dysphagia, or functional limitations typically require surgical debridement with careful preservation of flap vasculature. Drew *et al.* describe an intraoral surgical approach involving piecemeal bony resection under image guidance, combined with soft tissue interposition and physical therapy to additionally minimize recurrence and restore jaw function [9].

## Conclusion

Pedicle ossification following fibular free flap reconstruction is a rare but clinically significant complication when it results in bony bridging. Although most reported in adults, this case displays that pediatric patients especially those undergoing maxillary reconstruction are also at risk. Early recognition through imaging and prompt surgical intervention can restore function and prevent long-term morbidity.
